# Structure-based virtual identification of natural inhibitors of SARS-CoV-2 and its Delta and Omicron variant proteins

**DOI:** 10.2217/fvl-2022-0184

**Published:** 2023-06-01

**Authors:** Abdullah R Alanzi, Mohammad K Parvez, Mohammed S Al-Dosari

**Affiliations:** 1 Department of Pharmacognosy, College of Pharmacy, King Saud University, Riyadh, 11451, Saudi Arabia

**Keywords:** antivirals, Delta, helicase, Mpro, natural products, Omicron, SARS-CoV-2, Spike

## Abstract

**Aim:**

Structure-based identification of natural compounds against SARS-CoV-2, Delta and Omicron target proteins.

**Materials & methods:**

Several known antiviral natural compounds were subjected to molecular docking and MD simulation against SARS-CoV-2 Mpro, Helicase and Spike, including Delta and Omicron Spikes.

**Results:**

Of the docked ligands, 20 selected for each complex exhibited overall good binding affinities (-7.79 to -5.06 kcal/mol) with acceptable physiochemistry following Lipinski's rule. Finally, two best ligands from each complex upon simulation showed structural stability and compactness.

**Conclusion:**

Quercetin-3-acetyl-glucoside, Rutin, Kaempferol, Catechin, Orientin, Obetrioside and Neridienone A were identified as potential inhibitors of SARS-CoV-2 Mpro, Helicase and Spike, while Orientin and Obetrioside also showed good binding affinities with Omicron Spike. Catechin and Neridienone A formed stable complexes with Delta Spike.

## Plain language summary

Mutant strains of SARS-CoV-2 called ‘variants of concern’ (VOCs) are linked to a good ability to infect, re-infect and spread among people. They are also linked to poor ability to fight the disease and reduced effectiveness of vaccines. Delta and Omicron are important VOCs because they are difficult to control and treat. Specific resistance to some drugs used to treat COVID-19 poses a further challenge. Therefore, discovering natural or plant-derived drugs with no known resistance would be valuable to the treatment of COVID-19. In this study, we screen and identify seven plant-derived compounds that may be useful to treating COVID-19 – we identify Quercetin-3-acetyl-glucoside, Rutin, Kaempferol, Catechin, Orientin, Obetrioside and Neridienone A as potential candidates. Orientin, Obetrioside, Catechin and Neridienone A are identified as candidates against Delta and Omicron for the first time.

## Tweetable abstract

We report structure-based identification of natural compounds viz., Quercetin-3-acetyl-glucoside, Rutin, Kaempferol, Catechin, Orientin, Obetrioside and Neridienone A against SARS-CoV-2 Mpro, Helicase and Spike as well as Delta and Omicron Spike proteins.

The ongoing COVID-19 pandemic caused by SARS-CoV-2 has affected ∼760 million people, resulting in >6.8 million deaths worldwide [1–3]. SARS-CoV-2 is an enveloped virus with a plus-sense, single-strand RNA genome (∼29.9 kb) which codes for four structural and several nonstructural or accessory proteins [4,5]. Of its several nonstructural proteins, Mpro and Helicase play critical roles in RNA replication, productive infection, host-immune modulation and pathogenesis [5,6]. The structural protein Spike interacts with the host-cell receptor ACE2 through the receptor binding domain (RBD) of its subunit ‘S1’, while subunit ‘S2’ is involved in cell-membrane fusion during virus entry [6,7]. Therefore, Spike-RBD offers an essential target for developing effective drugs against SARS-CoV-2. However, the recent emergence of five Spike-RBD mutants (e.g., Alpha, Beta, Gamma, Delta and Omicron), called the ‘variants of concern (VOCs)’, has dramatically compromised disease control strategies [8–10]. Notably, Delta and Omicron have been directly implicated in enhanced ACE2-binding, high rate of infection/re-infection and transmission as well as immune-evasion or low vaccine efficiency leading to disease severity [9,10]. While some of the overlapping mutations in other VOCs have been investigated for ACE-2 binding affinity, transmissibility and immune-escape, the roles of other Spike mutations still remain unknown.

SARS-CoV-2 infection manifests as cough, fever, headache and breathlessness, which may progress to mild-to-fatal pneumonia especially in old age groups or those with comorbid pulmonary, cardiac, renal or hepatic disorders [3]. Over the two years of the COVID-19 pandemic, we have gained an outstanding knowledge of its epidemiology, clinical presentations, pathophysiology, immunology, treatments and preventive strategies. As a result of revolutionary approaches, several effective vaccines have been quickly developed and licensed to immunize the majority of the world's population. Moreover, in the absence of specific therapeutics, several repurposed drugs are currently in the final stages of human trials or have been granted approval for emergency use [10]. However, to the best of our knowledge, there is no approved drug targeting SARS-CoV-2-specific proteins. Delta and Omicron potentially challenge precautionary measures as well as diagnostic and treatment strategies that would facilitate the absolute control of COVID-19.

Natural products or plant secondary metabolites have high chemical diversity and biochemical-specificity with broad-spectrum pharmacological properties. Therefore, a wide range of bioactive compounds of different phytochemical classes such as flavonoids, saponins, steroids and tannins have been extensively reported for their remarkable antiviral properties against various DNA or RNA viruses [11]. Also, compared with natural therapeutic products, marketed antiviral drugs are too expensive for the majority of developing countries. Therefore, there has been always an urgent need to discover and develop novel natural antiviral agents with greater efficacy and safety, especially against emerging new pathogenic viruses. In view of this, many COVID-19 patients preferred traditional medicines, albeit using them together with conventional drugs. In a clinical study, for example, almost 90% of hospitalized COVID-19 patients in Chongqing, China were given herbal medicines along with lopinavir, ritonavir and interferon [12]. Further, based on *in silico* screening and *in vitro* bioassays, several natural or plant-derived compounds and their synthetic analogs have been confirmed to possess target-specific inhibitory effects against SARS-CoV-2 proteins [13–17]. Of these, Plitidepsin isolated from marine squirt has been shown to inhibit SARS-CoV-2 as well as Delta and Omicron replication *in vitro* [18]. Notably therein, the mechanism underlying the antiviral action of Plitidepsin is known to be mediated through inhibition of the host factor (eEF1A) rather directly targeting viral proteins. Nonetheless, to our best knowledge, no phytochemical has been reported for antiviral activity against Delta and Omicron. In this report, we have intended to identify potential natural or plant-derived compounds against SARS-CoV-2 as well as its VOCs Delta and Omicron, using structure-based *in silico* approaches.

## Materials & methods

### Ligand selection & preparation

For the screening of anti-SARS-CoV2 candidates, a range of natural compounds of different phytochemical classes known for antiviral activities were selected from the published literature. The ligands' 2D structures retrieved from PubChem (https://pubchem.ncbi.nlm.nih.gov/) were prepared in LigPrep of Maestro [19]. Their geometries were optimized and 30 conformers for each were generated, followed by their energy-minimization, using the OPLS_2005 forcefield [20]. For docking analysis, their finally prepared 3D structures were saved as ‘.mae’ files in Maestro Molecular Model Format [21].

### Protein preparation

For the structure-based screening of natural compounds, the crystal structures of SARS-CoV-2 target protein viz., Spike (PDB ID: 6M0J), Mpro (PDB ID: 6LU7) and Helicase (PDB ID: 6ZSL), including Spikes of Omicron (PDB ID: 7T9L) and Delta (PDB ID: 7V8B) variants were retrieved from Protein Data Bank (https://www.rcsb.org). Their 3D structures were prepared in the Protein Preparation Wizard of Schrodinger Maestro [21]. The protein structures were processed by adding hydrogen toms, disulfide and zero-order bonds to metals, assigning bond orders, as well as by deleting water molecules or any unwanted ligands. Further, the hydrogen bonds were optimized (pH 7.0) by using PROPKA [22], and energies were minimized (OPLS_2005 forcefield), followed by generating a 3D grid at the known binding site of each protein to assess site-specific ligand-target interactions.

### Molecular docking analysis

The docking of processed ligands on to the specific binding sites of the target proteins Mpro, Helicase and Spike (wild-type, Omicron variant and Delta variant) was performed using a glide docking module in standard precision (SP) mode [23]. The docked ligands were analyzed and selected on the basis of their estimated glide scores.

### Physicochemical properties prediction

The top selected inhibitor compounds were analyzed for their physicochemical properties, such as molecular weight (MW), hydrogen bond donors (HBD) and hydrogen bond acceptors (HBA), as well as the relationship between polar surface area (PSA) and octanol-water partition coefficient (logP) were predicted in QikProp of Maestro [24].

### Binding modes analysis

For all selected inhibitor compounds, their binding modes were analyzed and two inhibitors for each protein were selected on the basis of their best possible binding modes for protein–ligand complex stability.

### Molecular dynamic (MD) simulation

The docked complexes of SARS-CoV-2 Mpro, Helicase and Spike as well as Delta and Omicron Spike proteins for two selected compounds were evaluated for protein–ligand stability by MD Simulation at 50 ns, using VMD [25] and NAMD [26] tools. The input files of the complexes were prepared in Ambertools 21 [27], where the Antechamber was used to generate the ligand topology files and the missing hydrogens were supplemented by using LeaP program [28]. Each complex was further solvated in a solvation box (10 Å) containing TIP3P water model [29]. Moreover, counter Na^+^ and Cl^-^ ions were added to neutralize the systems, and were minimized to remove clashes in ff14SB and GAFF forcefields for target and ligand, respectively [30]. Finally, the complexes were subjected to additional equilibrations at 200, 250, and 300 K prior to simulation. The MD trajectories were saved at every 2 ps interval during the 50 ns period, and analyzed using CPPTRAJ [31] and R package [32].

## Results

### Molecular docking

The selected 49 compounds of diverse phytochemical classes docked to find their binding affinities with SARS-CoV-2 Mpro, Helicase and Spike, and Delta and Omicron Spike, were ranked according to their estimated glide scores, and of these, 20 compounds for each target protein were selected (Table 1). Theie binding-affinities (kcal/mol) ranged between -7.797 and -5.278 for Mpro; -6.982 and -5.657 for Helicase; -5.659 and -5.062 for Spike of SARS-CoV-2; -7.3063 and -5.586 for Spike of Omicron and, -6.221 and -5.285 for Spike of Delta.

**Table 1.** The virtual binding-affinities (kcal/mol) of the selected natural compounds (n = 49) against the respective target protein of SARS-CoV-2, Delta and Omicron.

Natural compound	Glide score (kcal/mol)				
	SARS-CoV-2 Mpro (PDB: 6LU7)	SARS-CoV-2 Helicase (PDB: 6ZSL)	SARS-CoV-2 Spike (PDB: 6M0J)	Omicron Spike (PDB:7T9L)	Delta Spike (PDB: 7V8B)
Kaempferol	-7.797	-5.657	-5.205	-5.584	-5.239
Quercetin	-7.342	-6.366	-5.116	^†^	-5.872
Catechin	-6.861	-6.172	-5.659	-6.039	-6.188
Luteolin	-6.737	^†^	^†^	^†^	-5.609
Rutin	-6.566	-6.293	-5.512	^†^	-5.635
Orientin	-6.179	-5.719	^†^	-6.727	^†^
Epicatechin	-5.913	-6.716	-5.335	-6.664	-5.985
Saponarin	-5.849	-5.808	^†^	-6.037	-5.521
Quercetin-3-acetyl-glucoside	-5.798	-6.762	-5.482	-6.495	^†^
Molnupiravir	-5.729	-6.135	-5.275	^†^	-6.221
3,4-dihydroxybenzoic acid	-5.686	^†^	^†^	^†^	^†^
Naringenin	-5.686	^†^	-5.097	^†^	^†^
p-hydroxybenzoic acid	-5.554	^†^	^†^	^†^	^†^
Chlorogenic acid	-5.548	^†^	^†^	^†^	^†^
Pinitol	-5.546	^†^	-5.062	^†^	-5.374
Hesperetin	-5.491	^†^	^†^	^†^	-5.607
Acacetin	-5.449	^†^	^†^	^†^	^†^
Carissanol	-5.349	^†^	^†^	^†^	^†^
Carinol	-5.333	-5.719	^†^	^†^	-5.606
Nortrachelogenin	-5.278	^†^	^†^	^†^	-5.107
Digitoxigenin	^†^	^†^	-5.541	^†^	^†^
Secoisolariciresinol	^†^	^†^	-5.472	-6.618	-5.377
Caffeic acid	^†^	^†^	-5.374	^†^	^†^
Sweroside	^†^	^†^	-5.271	^†^	^†^
Ritonavir	^†^	^†^	-5.236	-6.366	^†^
Quercetin-3-O-glucoside	^†^	-5.976	-5.236	-6.151	^†^
Liquiritin	^†^	-6.123	-5.221	-5.642	^†^
Isovitexin	^†^	-5.724	-5.125	-5.651	^†^
4–Epi-aubergenone	^†^	^†^	-5.125	^†^	^†^
Quercetin-3-O-pentoside	^†^	-5.898	-5.099	^†^	^†^
Indinavir	^†^	^†^	-5.096	-6.94	^†^
Obetrioside	^†^	-6.982	^†^	-7.303	^†^
Honghelotrioside	^†^	-6.116	^†^	^†^	^†^
Sitosterol glucoside	^†^	-6.094	^†^	^†^	^†^
Stigmasterol glucoside	^†^	-5.916	^†^	^†^	^†^
Methylswertianin	^†^	-5.725	^†^	^†^	^†^
Echujine	^†^	-5.718	^†^	-6.102	^†^
Obebioside C	^†^	^†^	^†^	-6.53	^†^
Ampelopsin F	^†^	^†^	^†^	-6.016	-5.382
Quercetin-3-acetyl rhamnoside	^†^	^†^	^†^	-6.003	^†^
β-Eudesmol	^†^	^†^	^†^	-5.844	^†^
Caffeic acid methyl ester	^†^	^†^	^†^	-5.616	^†^
2α-Carissanol	^†^	^†^	^†^	-5.586	^†^
Neridienone A	^†^	^†^	^†^	^†^	-6.0
Epicatechin gallate	^†^	^†^	^†^	^†^	-5.84
Acetylphenol	^†^	^†^	^†^	^†^	-5.68
Isofraxidin	^†^	^†^	^†^	^†^	-5.268
Lariciresinol	^†^	^†^	^†^	^†^	-5.08
Hongheloside C	^†^	^†^	^†^	^†^	-5.076

† Not significant.

### Physicochemical properties prediction

Of the ligands analyzed for their physicochemical properties, 37 best inhibitors were predicted for their MW, HBD, HBA, logP, and PSA (Table 2). Their predicted values were in acceptable ranges: MW (<500 g/mol), HBD/HBA (<10) and logP (<5). Notably, with few exceptions, all compounds complied with the criteria of Lipinski's rule of five.

**Table 2.** The virtual determination of physicochemical properties of the selected antiviral natural compounds (n = 37).

No.	Natural compounds	MW (g/mol)	HBD	HBA	QPlogP_o/w_	PSA	RO5 (violations)
1	Kaempferol	286.24	3	5	1.061	121.15	0
2	Quercetin	302.24	4	5	0.385	142.683	0
3	Catechin	290.272	5	5	0.479	115.741	0
4	Luteolin	286.24	3	5	0.948	121.176	0
5	Rutin	610.524	9	21	-2.695	270.732	3
6	Orientin	448.382	7	13	-1.318	207.873	2
7	Epicatechin	290.272	5	5	0.491	115.46	0
8	Saponarin	594.525	9	21	-2.494	264.21	3
9	Quercetin-3-acetyl-glucoside	464.382	7	14	-1.463	219.83	2
10	Molnupiravir	329.309	4	13	-1.793	164.402	1
11	3,4-dihydroxybenzoic acid	154.122	3	4	0.031	93.524	0
12	Naringenin	272.257	2	4	1.653	100.299	0
13	p-hydroxybenzoic acid	138.123	2	3	0.585	72.006	0
14	Chlorogenic acid	354.313	6	10	-0.25	185.475	1
15	Pinitol	194.184	5	10	-1.856	110.809	0
16	Hesperetin	302.283	2	5	1.803	107.691	0
17	Acacetin	284.268	1	4	2.48	85.391	0
18	Carissanol	252.353	2	4	2.039	58.82	0
19	Carinol	378.421	5	7	1.981	118.465	0
20	Nortrachelogenin	374.39	3	7	2.381	113.46	0
21	Digitoxigenin	374.519	2	5	3.082	80.343	0
22	Secoisolariciresinol	362.422	4	6	2.44	101.308	0
23	Caffeic acid	180.16	3	3	0.557	95.637	0
24	Sweroside	358.344	4	14	-1.599	147.53	1
25	Ritonavir	720.943	3	11	5.909	146.377	2
26	Quercetin-3-O-glucoside	464.382	7	14	-1.463	219.83	2
27	Liquiritin	418.399	5	13	-0.211	159.648	1
28	Isovitexin	432.383	6	12	-0.56	185.794	2
29	4–Epi-aubergenone	236.353	1	2	2.993	46.722	0
30	Quercetin-3-O-pentoside	434.356	6	12	-0.846	192.056	2
31	Indinavir	613.798	4	14	2.655	121.149	2
32	Obetrioside	917.009	9	31	-1.771	311.595	3
33	Honghelotrioside	901.01	8	30	-1.005	287.751	3
34	Sitosterol glucoside	576.855	4	10	5.233	99.491	1
35	Stigmasterol glucoside	574.84	4	10	5.03	100.597	1
36	Methylswertianin	288.256	0	4	2.504	89.21	0
37	Echujine	842.973	8	28	-0.997	256.29	3

HBD: Hydrogen-bond donor; HBA: Hydrogen-bond acceptor; MW: Molecular weight; PSA: Polar surface area; RO5: Rule of five.

### Binding pose prediction

**Figure 1.**
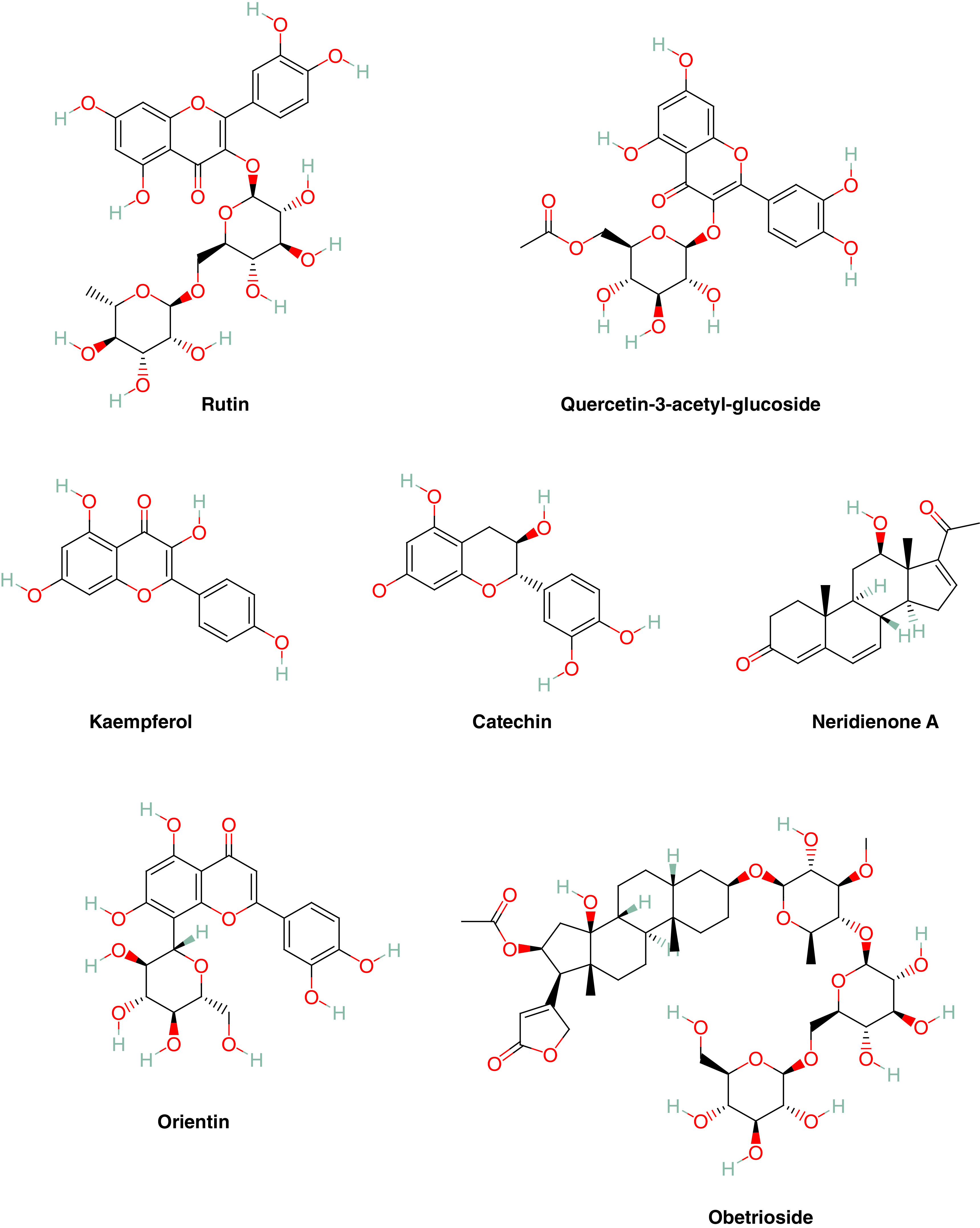
Chemical structures of the most active natural compounds (https://pubchem.ncbi.nlm.nih.gov/) selected by molecular docking against SARS-CoV-2, Delta and Omicron proteins.

**Figure 2.**
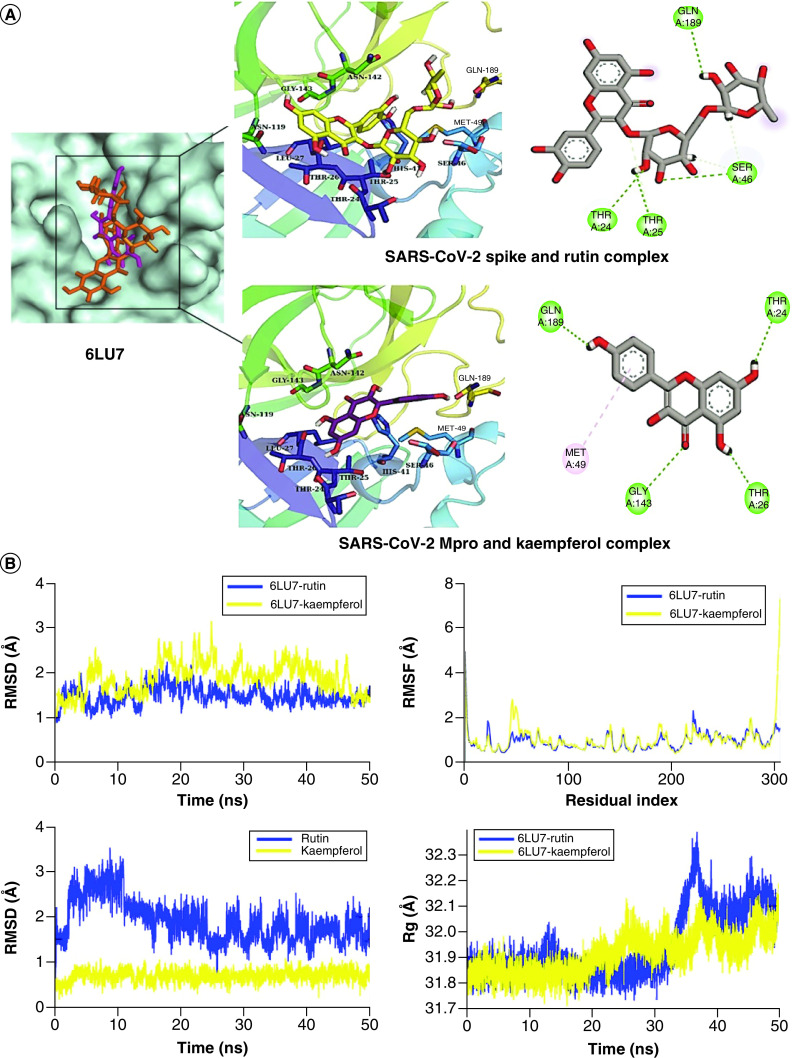
Structure-based virtual analysis of ligand–protein interaction. **(A)** Molecular docking showing the binding interactions of SARS-CoV-2 Mpro with Rutin and Kaempferol. **(B)** The interactions are shown in different colors. Hydrogen bonding (green), hydrophobic (magenta), Pi-Cation (orange). Molecular dynamics simulation showing the protein–ligand stability analysis (root mean square deviation and root mean square fluctuation) of SARS-CoV-2 Mpro with Rutin and Kaempferol.

**Figure 3.**
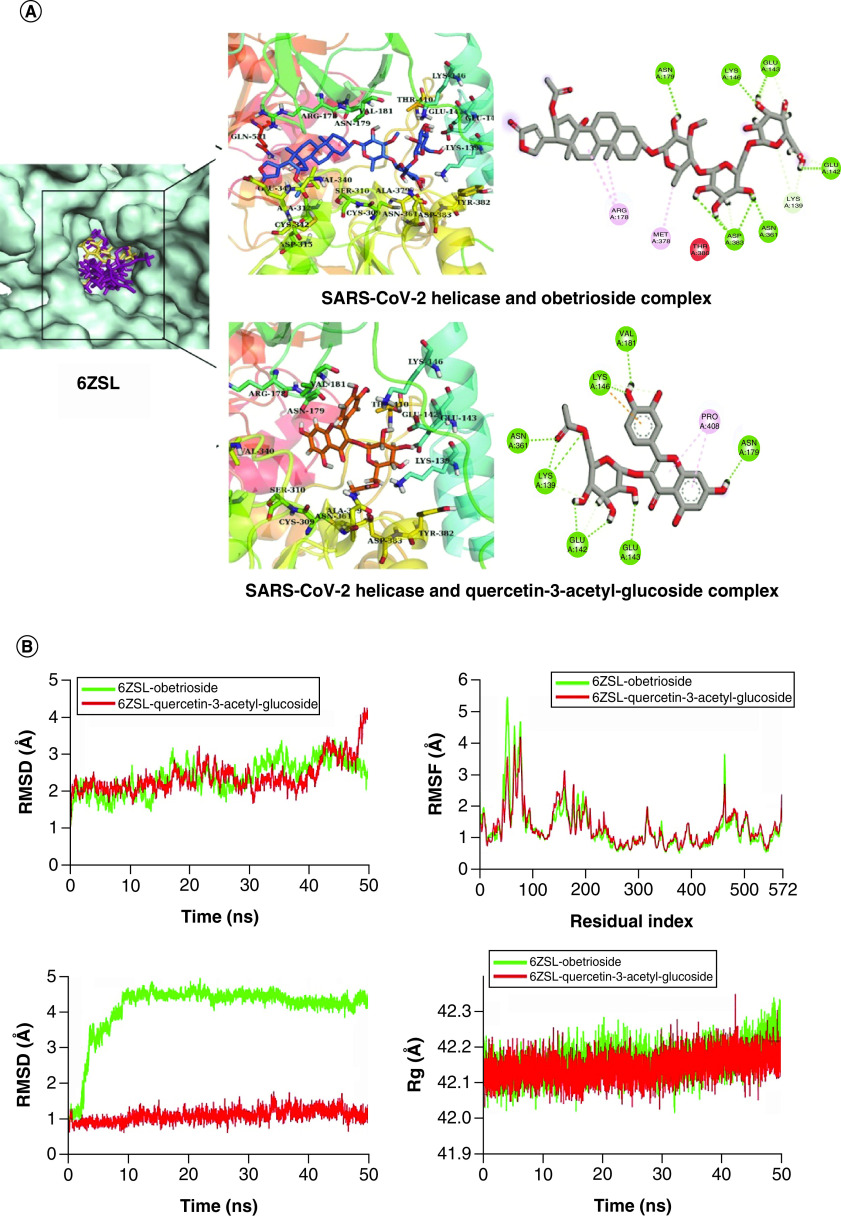
Structure-based virtual analysis of ligand–protein interaction. **(A)** Molecular docking showing the binding interactions of SARS-CoV-2 Helicase with Quercetin-3-acetyl-glucoside and Obetrioside. **(B)** The interactions are shown in different colors. Hydrogen bonding (green), hydrophobic (magenta), Pi-Cation (orange). Molecular dynamics simulation showing the protein–ligand stability analysis (RMSD and RMSF) of SARS-CoV-2 Helicase with Obetrioside and Quercetin-3-acetyl-glucoside.

Of the analyzed binding poses of the seven best-docked ligands (Figure 1), two from each complex were selected for assessing the protein–ligand stability. For the SARS-CoV2 Mpro, the selected ligands were Kaempferol and Rutin (Figure 1), whose interactions and binding poses are shown (Figure 2A). It was observed that Kaempferol formed four hydrogen bonds with MPro residues Thr24, The26, Gly143, and Gln189 whereas it also participated in Pi-alkyl interaction with Met49. Similarly, while Rutin formed hydrogen bonds with MPro residues Thr24, thr25, Ser46, and Gln189, it was not involved in any hydrophobic interaction. The binding poses showed that these ligands occupied the same space in the binding pocket of MPro (Figure 3A).

For Helicase, Obetrioside and Quercetin-3-acetyl-glucoside (Figure 1) were selected. Therein, Obetrioside formed 8 hydrogen bonds with Helicase residues Glu142, Glu143, Lys146, Asn179, Asn361, and Asp383 while interacted through Pi-Alkyl bonds with Arg178 and Met378. The similar residues were also involved in hydrogen bonding with Quercetin-3-acetyl-glucoside as well with an additionally Pi-Cation interaction with Lys145. Their overall binding modes described that these have similar poses in the binding pocket of Helicase (Figure 3A).

**Figure 4.**
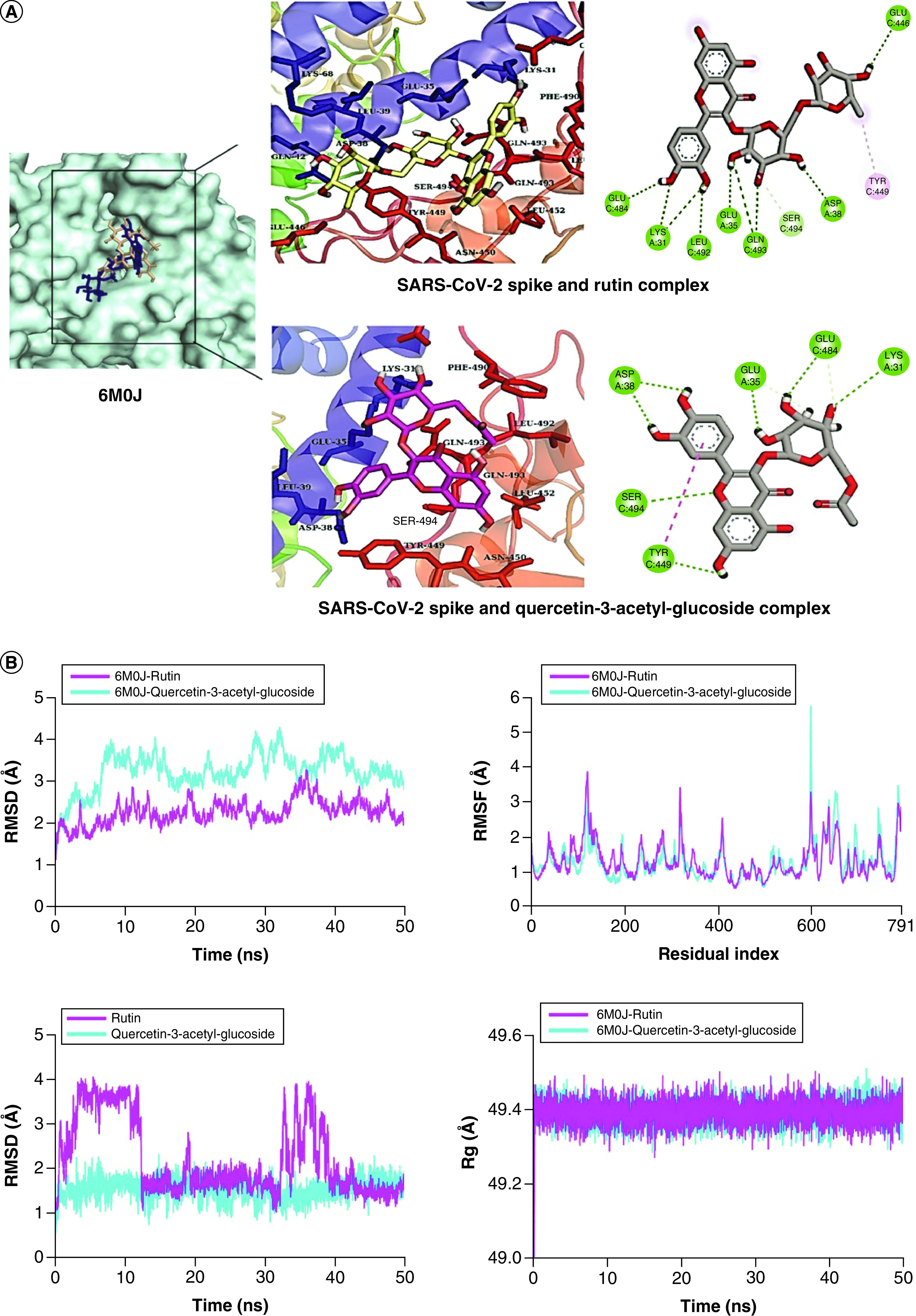
Structure-based virtual analysis of ligand–protein interaction. **(A)** Molecular docking showing the binding interactions of SARS-CoV-2 Spike with Rutin and Quercetin-3-acetyl-glucoside. **(B)** The interactions are shown in different colors. Hydrogen bonding (green), hydrophobic (magenta), Pi-Cation (orange). Molecular dynamics simulation showing the protein–ligand stability analysis (root mean square deviation and root mean square fluctuation) of SARS-CoV-2 Spike with Rutin and Quercetin-3-glusoside.

For SARS-CoV-2 Spike protein, Rutin and Quercetin-3-acetyl-glucoside (Figure 1) were selected. Rutin formed nine hydrogen bonds with Spike residues Lys31, Glu35, Asp38, Glu446, Glu484, Leu492, and Gln493, as well as also formed a Pi-Alkyl interaction with Tyr449. Contrarily, Quercetin-3-acetyl-glucoside formed 8 hydrogen bonds with Spike residues Lys31, Glu35, Asp38, Tyr449, Glu484, and Ser494 including a pi-pi stacking with tyr449. The binding poses of these two compounds were similar, too (Figure 4A).

**Figure 5.**
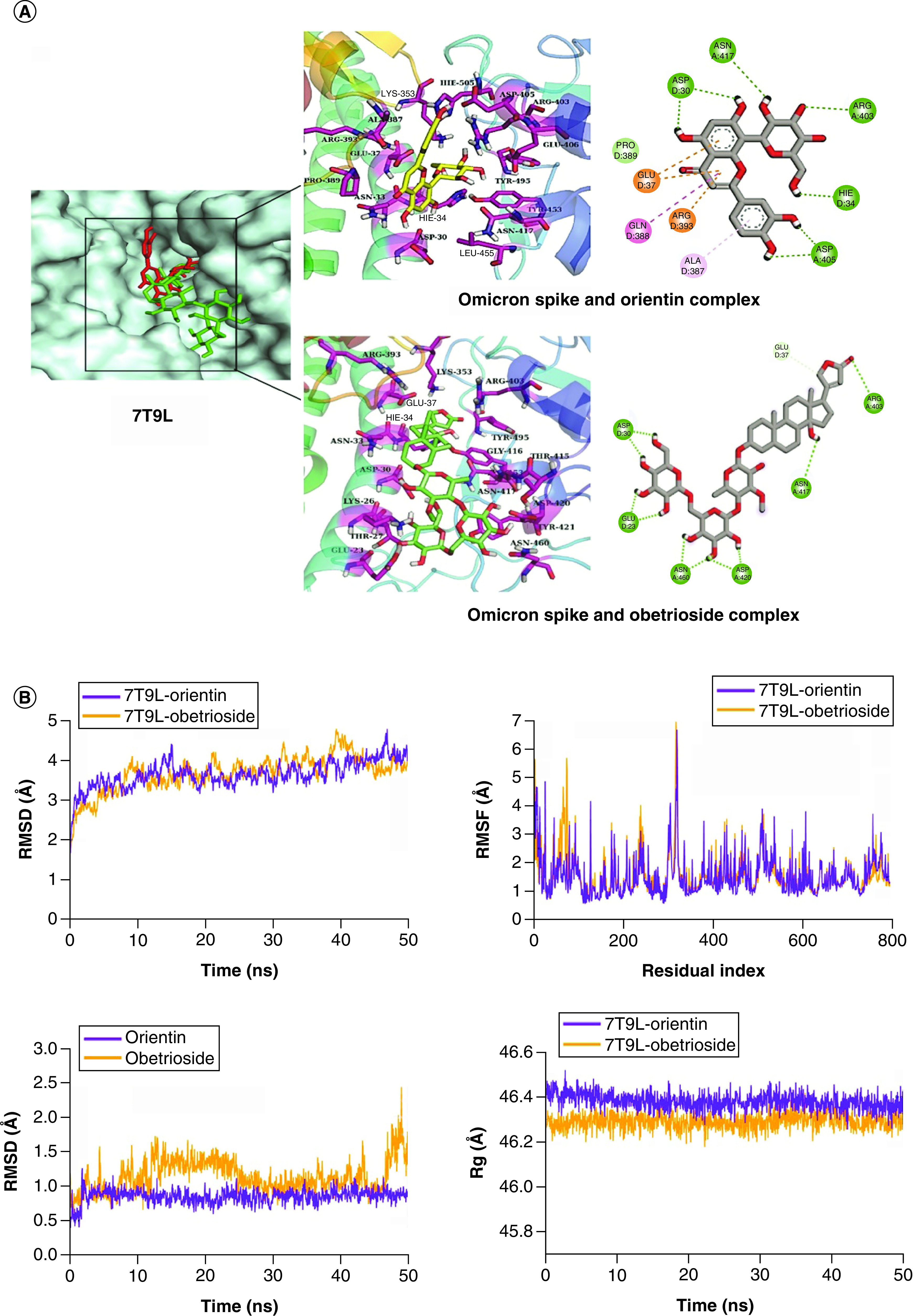
Structure-based virtual analysis of ligand–protein interaction. **(A)** Molecular docking showing the binding interactions of Omicron Spike with Orientin and Obterioside. **(B)**The interactions are shown in different colors. Hydrogen bonding (green), hydrophobic (magenta), Pi-Cation (orange). Molecular dynamics simulation showing the protein–ligand stability analysis (root mean square deviation and root mean square fluctuation) of Omicron Spike with Orientin and Obetrioside.

In the case of Omicron Spike, Obetrioside and Orientin (Figure 1) were selected. Therein, Obetrioside formed 10 hydrogen bonds with Glu23, Asp30, Arg403, Asn417, Asp420 and Asn460 residues. Orientin formed hydrogen bonding with Asp30, His34, Arg403, Asp405, and Asn417 residues as well as showed Pi-Alkyl interaction with Gln388 and Pi-cation interactions with Glu37 and Arg393. Both Obetrioside and Orientin occupied the same space in the binding pocket of the Omicron Spike (Figure 5A).

**Figure 6.**
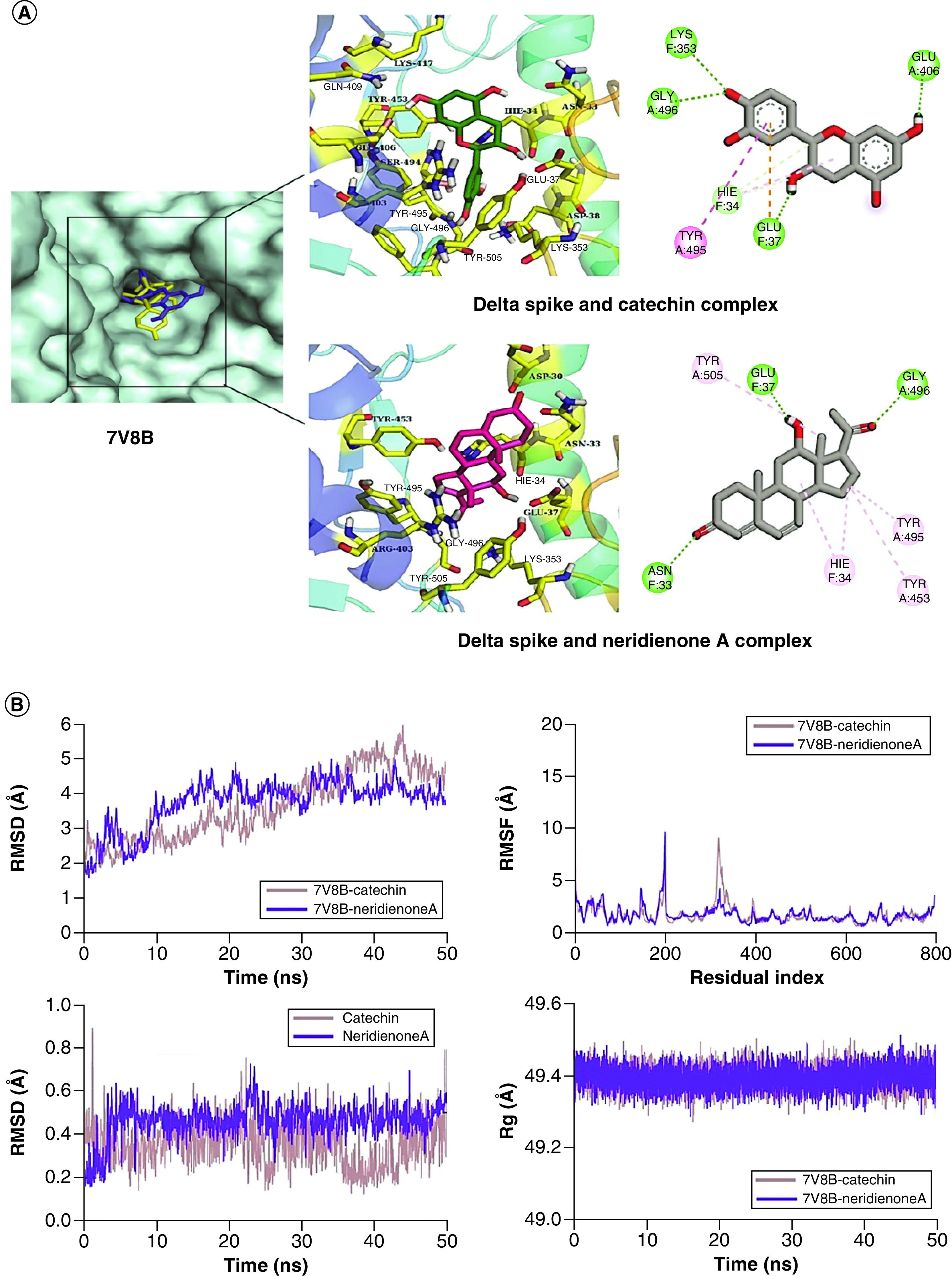
Structure-based virtual analysis of ligand–protein interaction. **(A)** Molecular docking showing the binding interactions of Delta Spike with Catechin and Neridienone A. **(B)** The interactions are shown in different colors. Hydrogen bonding (green), hydrophobic (magenta), Pi-Cation (orange). Molecular dynamics simulation showing the protein–ligand stability analysis (root mean square deviation and root mean square fluctuation) of Delta Spike with Catechin and Neridienone A.

Lastly, for Delta Spike, Catechin and Neridienone A (Figure 1) were selected. Therein, Catechin formed four hydrogen bonds with Delta Spike residues Glu37, Lys353, Glu406 and Gly496, including one Pi-Alkyl interaction with Tyr495. In contrast, Neridienone A was involved in three hydrogen bonds with Asn33, Glu37, and Gly496, as well as showed hydrophobic interactions with His34, Tyr453, Tyr495 and Tyr505 (Figure 6A).

### MD simulation

The MD trajectories of all selected ligand–protein complexes were analyzed to estimate their RMSD, RMSF, and Rg, separately. The RMSD (∼1.5 Å) of Mpro complexed with Rutin and Kaempferol showed their attainment of equilibrium at 5 ns (Figure 2B). After equilibration, the Mpro and Rutin complex did not show any major deviation in the RMSD value while Mpro and Kaempferol complex displayed major deviations between 15 and 30 ns where the value increased to ∼2.5–3 Å but lately, regained its initial conformation as Mpro and Rutin complex. The RMSD plots exhibited that Kaempferol was bound stably with Mpro with no deviation in RMSD value while Rutin showed some major deviations during the first 15 ns and then acquired stability (∼1.5–2.5 Å). Similarly, the RMSF analysis showed no major fluctuations in Mpro residues. Therein, while the higher RMSF showed the loops region, the lower value indicated the rigid secondary structure of the protein. The Mpro residues did not display major fluctuations except for C- and N-terminals. The Rg value represents the compactness of protein structure, where higher score indicated protein's unfolding events, if any, during simulation. The Rg plots of Mpro complexes indicated both systems stability until 30 ns, but then a deviation of ∼0.5 Å was seen in Mpro and Rutin complex during 30–40 ns, which attained stability after 40 ns at ∼32.1–32.2 Å. Contrarily, the Mpro and Kaempferol complex demonstrated a deviation of ∼0.2 Å during this phase. Overall, Mpro in the complex did not encounter any unfolding event during the simulation and remained compact.

The complexes of Helicase with Obetrioside and quercetin-3-glucoside were selected for simulation. Therein, the RMSD plots of Helicase backbone atoms showed their RMSD values in the range of ∼2–2.5 Å from 1 to 20 ns, which later slightly increased to ∼3 Å at 20 ns. The RMSD values deviated from ∼2–3 Å between 20 and 40 ns. toward the end of the simulation, the RMSD of Helicase and quercetin-3-glucoside complex deviated to ∼4 Å while the Helicase and Obetioside complex attained the initial conformation. The RMSD analysis of ligands showed that quercetin-3-glucoside remained stable at ∼1 Å till the end but Obetrioside showed a major conformational change during the first 10 ns and then attained stability at ∼4.5 Å without any further changes (Figure 3B). The RMSF showing major fluctuations in the residues from 50 to 100 indicated this region with loops. Some minor fluctuations in the residues from ∼140 to 180 were also observed but the remaining residues did not show any notable fluctuation. Similarly, the Rg values showed that Helicase bound to either ligand remained compact as the Rg values did not show major variations and remained between ∼42–42.5 Å during the whole simulation time. This indicated that the two ligands did not induce any unfolding event in the Helicase structure.

In the case of SARS-CoV-2 Spike protein, its complexes with Rutin and Quercetin-3-glusoside were selected for MD simulation analysis. The RMSD of backbone atoms of SARS-CoV-2-Spike and Rutin complex did not show major deviations as it remained in the range of ∼2–3 Å all the time. In contrast, the RMSD of SARS-CoV-2 Spike and quercetin-3-glucoside complex deviated to ∼4 Å at 10 ns and stayed in the range of ∼3–4 Å till 40 ns, and then decreased to ∼3 Å toward the end (Figure 4B). While the RMSD of quercetin-3-glucoside was thoroughly stable, Rutin displayed major deviations in the first 10 ns and then between 35 to 40 ns in their respective complexes. The RMSF analysis indicated no major fluctuation in SARS-CoV-2 Spike residues. Though some minor fluctuations were seen, the overall structure did not show flexibility. Similarly, the Rg values showed the protein compactness when bound to both ligands during the simulation.

For the Omicron Spike protein, Orientin and Obetrioside were selected for stability analysis. The RMSD of Omicron Spike backbone showed that the complexes were equilibrated at 5 ns, and remained in the range of ∼3–4 Å till 40 ns. Later, the RMSD of Omicron Spike and Obetrioside increased to ∼5 Å but it attained the previous confirmation at 45 ns and remained in the same range till the end. However, under the same conditions, the RMSD of Omicron Spike and Orientin showed a small deviation at 45 ns (Figure 5B). The RMSD of Orientin was stable throughout the simulation in the range of ∼0.75 to 1 Å while Obetrioside showed deviations but it was stable as the RMSD remained in the range of >2 Å. The RMSF analysis showed that the Omicron Spike residues did not show major fluctuations in both complexes except for loop regions. A minor fluctuation in the Omicron Spike and Obetrioside complex was observed in the residues ∼70 to 80. The Rg analysis showed that while the Omicron Spike and Obetrioside complex remained in the range of ∼46.2 to 46.3 Å, Omicron Spike and Orientin complex remained in the range of ∼46.4 Å. The Rg plots of both complexes showed that the protein remained compact during the simulation and did not show any conformational changes.

In order to assess the stability of Delta Spike protein, its complexes with Catechin and Neridienone A were analyzed. The RMSD plots of both complexes showed that they attained equilibrium at 5 ns, which were then observed to be <3 Å till 10 ns and then underwent deviations. The RMSD of Delta Spike and Catechin increased to ∼4 Å at 30 ns, and then deviated in the range of 3 to 4 Å till the end. In contrast, the RMSD of Delta Spike and Neridienone A decreased to 2 Å after 10 ns and remained in the range of ∼2 to 3 Å till 40 ns, and then decreased to ∼1.75 Å toward the end of the simulation (Figure 6B). Similarly, the RMSD plots of both ligands showed that they were thoroughly stable as the RMSD values were <0.6 Å during simulation. The RMSF plots of both complexes showed that the Delta Spike residues did not show major fluctuations except for few form ∼190 to 200 and ∼300 to 320. The remaining residues showed the rigid behavior. The Rg analysis of both complexes showed that the protein was compact during the simulation as their Rg values were in the range of ∼49.3 to 49.5 Å.

## Discussion

Molecular docking as well as MD simulation analysis has emerged as a crucial *in silico* tool that utilizes bioinformatics programs to closely predict how a small molecule (ligand) interacts with a receptor protein (target) toward identifying new therapeutics [33]. Furthermore, they accurately determine ‘ligand–protein’ interactions, and help establish the ‘structure–activity relationship (SAR)’ of a promising compound [19]. Therefore, based on the docking and SAR analysis, several natural compounds and their synthetic analogs have been confirmed to possess target-specific inhibitory effects against SARS-CoV-2 proteins [13–18]. The SARS-CoV-2 nonstructural gene (*Nsp5*) codes for 3CLpro or Mpro which by hydrolyzing the replicase polyprotein (nsp1), produces Nsp4-Nsp16 components, and thus plays a crucial role in its RNA replication and the maturation of nonstructural proteins [5,6,10]. Notably, due to a highly conserved protein and the absence of its human homolog, 3CLpro is one of the most researched drug targets for SARS-CoV-2. The most studied 3CLpro targeting drugs are ‘peptide-mimetic’ inhibitors similar to its natural peptide substrate, and it is also the most studied at present [6,10]. Further, the nonstructural gene (Nsp13) encodes the multi-functional enzyme with Helicase, GTPase and ATPase activities, essentially involved in RNA unwinding and 5′mRNA capping during the replication cycle [5,6,10]. SARS-CoV-2 Nsp13 is, therefore, an important drug target. In previous *in silico* studies, several inhibitor molecules against Mpro have been identified [14,15]. Notably, significant mutations in the SARS-CoV-2 Spike-RBD, including others have dramatically changed its transmissibility and pathogenicity [34]. The RBD mutations potentially contribute to the emergence of its globally recognized VOCs with high rates of infectivity or re-infectivity, transmissibility, neutralization resistance to natural immunity or vaccination and therefore, the severity of COVID-19 [8,35,36]. Recent studies have reported that these VOCs have 45–71% more transmissibility than SARS-CoV-2 [36]. Of these, the notable RBD mutations are ‘T478K’ in Delta and Omicron; ‘N501Y’ in Alpha, Beta, Gamma and Omicron; ‘K417N/E484K’ in Beta, Gamma and Omicron. In addition, mutations in the flanking regions of RBD such as ‘D614G’ in Alpha, Beta, Gamma, Delta and Omicron have been also implicated in enhanced infectivity and disease severity [35–37].

Delta remains the dominant VOC with three RBD mutations (L452R, T478K, and E484K), including others, and has been directly implicated in increased infectivity, transmission and immune escape [6,10]. Omicron, with higher rates of transmissibility, infection or re-infection and immune escape ability, has over 50 mutations of which at least 16 are in the RBD [6,10]. While some of the overlapping mutations in other VOCs have been investigated for ACE-2 binding-affinity, transmissibility and immune-escape, the roles of other mutations still remain unknown.

Notably, several of the tested antiviral natural compounds belonging to different phytochemical classes have been further confirmed for their broad-spectrum activities against many pathogenic DNA and RNA viruses [11,38]. Also, antiviral drug resistance related to some specific gene mutations in RNA viruses due to prolonged therapy remains a clinical challenge. Fortunately, there is no published report on natural antiviral-associated resistance in any such studied viruses. In addition, the emergence of immune/vaccine escape variants of several viruses, especially novel viruses like SARS-CoV-2 due to amino acid mutations in the antigenic epitope(s) has hampered the efficacies of available vaccines. Omicron, including its sub-lineage, has been questioned for its association with compromised protection in vaccinated populations or productive re-infection worldwide [10]. This has opened new frontiers to check the efficiency of approved vaccines against the pathogenic Delta and Omicron including other VOCs. Taken together, identifying and developing potential natural antiviral agents would be a valuably better fit for the treatment and control of COVID-19. In the present study, therefore, we have selected natural bioactive compounds reported for their potential antiviral efficacies. We have utilized *in silico* molecular docking and MD simulation to identify new potential natural compounds of diverse phytochemical classes against SARS-CoV-2 proteins. Prior to docking, the performance of the Glide tool was validated by docking of the reference ligand i.e., the available co-crystal structure of SARS-CoV2 Mpro was used. Mpro was extracted and docked to the same binding site followed by MD simulation where RMSD (0.22) of the complex was calculated. Notably therein, minor deviations showed the better performance of the Glide tool.

Of the 37 ligands docked against Mpro, Helicase and Spike of SARS-CoV-2 as well as Spikes of both Omicron and Delta (VOCs), twenty compounds for each target were selected. Their overall binding affinities in the complexes were in the range of -7.797 to -5.062 kcal/mol. Also, their predicted physiochemical properties like MW, logP and PSA etc. were found to be in the acceptable range, and they followed Lipinski's rule of five. For Mpro, Kaempferol and Rutin formed hydrogen bonds with common residues Thr24 and Gln189, including others in the binding pocket. For Helicase, Obetrioside and Quercetin-3-acetyl-glucoside formed hydrogen bonds with Glu142, Glu143, Lys146, Asn179, Asn361 and Asp383 as well as interacted through Pi-Alkyl bonds with Arg178 and Met378. For the SARS-CoV-2 Spike, Rutin and Quercetin-3-acetyl-glucoside formed hydrogen bonds with common residues Lys31, Glu35, Asp38, Glu484, and Gln493, as well as also showed a Pi-Alkyl interaction with Tyr449. In the case of Omicron Spike, Obetrioside and Orientin formed hydrogen bonds with common residues Asp30, Arg403, including others as well as showed Pi-Alkyl and Pi-cation interactions. For Delta Spike, Catechin and Neridienone A formed hydrogen bonds with common residues Glu37, including other interactions. Further, the RMSD and RMSF data of the selected ligand–protein complexes showed overall structural stability and compactness despite little fluctuation in few cases during MD simulation.

Natural flavonoids like Quercetin, Rutin and Kaempferol, including their derivatives have been reported for their antiviral activity against several RNA and DNA viruses including herpes simplex virus, cytomegalovirus, poliovirus, para-influenza virus, dengue virus, hepatitis B virus, respiratory syncytial virus, SARS-CoV-1 and SARS-CoV-2 [38–42]. Quercetin has been shown to suppress SARS-CoV-2 replication, whereas its synthetic analog 8-(*p*-tolylselenyl)-quercetin inhibited the activity of the SARS-CoV-2 Mpro *in vitro* [43]. The MD analysis of the complex of analog with Mpro showed a strong binding affinity and binding interaction of quercetin moiety with the Mpro catalytic residues. Moreover, Quercetin potently inhibited the SARS-CoV-2 Helicase RNA unwinding activity *in vitro*, as well as MD analysis showing its good binding affinity [44]. Interestingly, in a recent clinical trial, ‘Quercetin phytosome’ treatment of patients with mild COVID-19, significantly reduced viral load and improved serum biochemistry [45]. In the present study, we show the strong binding affinity of Quercetin-3-acetyl-glucoside with both Helicase and Spike of SARS-CoV-2.

Rutin (quercetin-3-rutinoside), a derivative of Quercetin has been reported for its antiviral activities against several viruses, including hepatitis B virus and SARS-CoV-2 proteins [39,40,46,47]. In a previous study, Rutin has been shown to interact with Mpro residues Thr26, Tyr54, Leu141, Glu166 and several others through hydrogen bonds, including pi-cation and pi-alkyl interactions involving His41 and Met49, respectively with showing high docking scores [48,49]. Moreover, in a virtual screening study, Rutin has been also predicted as a potential Helicase inhibitor with high mfScores [50]. Interestingly, a Chinese herbal formulation ‘Lianhuaqingwen’ with high Rutin content has been reported to treat COVID-19 patients [51]. In lie with this, we show strong binding affinity of Rutin with both Mpro and Spike of SARS-CoV-2.

Kaempferol and its derivatives has been reported to have broad antiviral activities against influenza virus, Japanese encephalitis virus, dengue virus, human immunodeficiency virus, hepatitis B virus and coronaviruses [39,40,52]. For SARS-CoV-2, several *in silico* studies have shown strong binding of Kaempferol with Mpro [52,53]. In lie with this, we also show strong binding affinity of Rutin with Mpro but not with other proteins of SARS-CoV-2.

Catechin is a flavonoid which along with its derivatives like epicatechin-3-gallate and epigallocatechin-3-gallate are known for potent antiviral activities against human immunodeficiency virus, herpes simplex virus, human T-cell leukemia virus, influenza virus, rotavirus, Epstein-Barr virus, and adenovirus as well as hepatitis B and hepatitis C viruses [54,55]. In addition, an MD simulation study has shown strong interaction of theaflavin 3-gallate with Mpro active site residues [56]. Further, a very recent *in silico* study of epigallocatechin gallate has shown strong interaction with Spike binding pocket residues [57]. Interestingly, our analysis has shown a good binding score of catechin with the Spike of Delta.

Orientin is a flavonoid, which has been shown as an influenza virus endonuclease inhibitor [58]. Very recently, *in silico* studies have revealed its weak binding affinity with SARS-CoV-2 Mpro [59,60]. Here we for the first time, predict a good binding affinity of Orientin with Spike of Omicron.

Obetrioside is a steroidal glycoside, and to our best knowledge, no bioactivity has been reported so far except its identification in *Adenium obesum* with cytotoxic activity [61]. In the present structure-based virtual analysis, we for the first time report a good binding affinity of Obetrioside with Helicase of SARS-CoV-2 as well as Spike of Omicron.

Neridienone A is a pregnane, previously reported for its anti-cell proliferative [62] and leishmanicidal [63] activities. However, to the best of our knowledge, its antiviral activity remains unknown. In this *in silico* analysis, we for the first time report a good binding affinity of Neridienone A with Spike of SARS-CoV-2 as well as Delta.

Furthermore, the human respiratory tract and gut are the primary habitats of microbiota with immunomodulatory potential in various diseases, including some viral infections [64]. Therein, microbiota-induced metabolites or other factors mediate microbes-host interactions toward further modulating the immune system. In some COVID-19 patients, significantly altered respiratory and gut microbiota have been linked to reduced proportions of probiotics as well as increased risk of opportunistic pathogens and inflammatory-cytokine storms [64]. Though the exact antiviral mechanism(s) of probiotics is poorly known, the potential antiviral effect of probiotic bacteria has been suggested as reinforcing the mucosal innate and system-acquired immune responses via anti-inflammatory effects. Therefore, several microbiota-based prophylaxes and therapeutic modules, including probiotics and prebiotics have been suggested. Notably, supplementation with prebiotics such as dietary fibers and nutraceuticals rich in bioactive phytochemicals (e.g., flavonoids, tannins, phytosterols) have been shown to be promising in treating COVID-19 patients [65,66]. In view of this, our suggested antiviral natural compounds would also contribute as effective probiotics against SARS-CoV-2.

## Conclusion

Our molecular docking and MD simulation analyses suggest seven bioactive phytochemicals Quercetin-3-acetyl-glucoside, Rutin, Kaempferol, Catechin, Orientin, Obetrioside and Neridienone A as potential direct-acting antiviral candidates against SARS-CoV-2 Mpro, Helicase and Spike proteins. Interestingly therein, while Orientin and Obetrioside also showed good binding-affinities with Omicron Spike, Catechin and Neridienone A formed stable complexes with Delta Spike. Notably, because no natural or plant-derived compound is known for direct antiviral activity against the VOCs, our data for the first time, proposes Orientin, Obetrioside Catechin and Neridienone A as potential antiviral candidates against Delta and Omicron. This, however, needs further studies, including experimental validations. Moreover, because there is no evidence of natural drug-associated viral resistance, discovering potential natural antivirals would be valuable for the control of VOCs like Delta and Omicron.

**Summary points**

- The recent emergences of SARS-CoV-2 variants of concern (VOCs) attributed to high infectivity or re-infectivity, transmissibility and diminished immune protection remain a challenge.
- Of the VOCs, Delta and Omicron have potentially disrupted the precautionary measures, diagnostic and treatment strategies toward the absolute control of the disease.
- Further, antiviral drug-resistance related to some specific mutations in RNA viruses due to prolonged use of conventional drugs is another problem in virus control.
- Together, this has opened new frontiers to evaluate the efficiency of approved vaccines and to identifying new drug candidates against Delta, Omicron and other VOCs.
- Therefore, discovering potential natural antiviral agents with no known drug-resistance would be valuable for the control of VOCs like Delta and Omicron.
- Here, we used molecular docking and molecular docking (MD) simulation to identify potential antiviral natural compounds targeting SARS-CoV-2, Delta and Omicron proteins.
- Of the 37 docked ligands, 20 for each complex exhibited overall good binding affinities with acceptable physiochemistry and Lipinski's rule.
- Quercetin-3-acetyl-glucoside, Rutin, Kaempferol, Catechin, Orientin, Obetrioside and Neridienone A appeared as potential candidates for SARS-CoV-2 Mpro, Helicase and Spike.
- Interestingly, while Orientin and Obetrioside also showed good binding affinities with Omicron Spike, Catechin and Neridienone A formed stable complexes with Delta Spike.
- Notably, compared with SARS-CoV-2, there is no natural or plant-derived compound reported for direct-acting antiviral efficacy against VOCs.
- Therefore, we for the first time, propose Orientin, Obetrioside Catechin and Neridienone A as potential antiviral candidates against Delta and Omicron.
- This, however, needs further studies, including experimental validations.
